# Flexible Arbeitszeitmodelle in der Rheumatologie: Ergebnisse einer deutschlandweiten Umfrage

**DOI:** 10.1007/s00393-025-01761-6

**Published:** 2025-12-08

**Authors:** Ann-Christin Pecher, Katinka Albrecht, Xenofon Baraliakos, Johanna Callhoff, Eugen Feist, Isabell Haase, Michaela Koehm, Martin Krusche, Philipp Sewerin, Anna Voormann, Sarah Ohrndorf, Johanna Mucke

**Affiliations:** 1 Medizinische Klinik II, Hämatologie, Onkologie, klinische Immunologie und Rheumatologie, Universitätsklinikum Tübingen, Tübingen, Deutschland; 2 Deutsches Rheuma-Forschungszentrum (DRFZ) Berlin, Programmbereich Epidemiologie und Versorgungsforschung, Berlin, Deutschland; 3 Rheumazentrum Ruhrgebiet, Ruhr-Universität Bochum, Claudiusstraße 45, 44649 Herne, Deutschland; 4 Magdeburg und Klinik für Rheumatologie und Klinische Immunologie, Experimentelle Rheumatologie der Otto-von-Guericke, Vogelsang-Gommern, Deutschland; 5 Sektion für Rheumatologie und Entzündliche Systemerkrankungen in der III. Medizin, Universitätsklinikum Hamburg-Eppendorf (UKE), Hamburg, Deutschland; 6 Rheumatologie, Immunologie – Entzündungsmedizin, Universitätsmedizin Goethe-Universität Frankfurt am Main, Fraunhofer Institut für Translationale Medizin & Pharmakologie ITMP & Fraunhofer Cluster of Excellence Immune Mediated Diseases CIMD, Frankfurt am Main, Deutschland; 7 Deutsche Gesellschaft für Rheumatologie und klinische Immunologie e. V., Berlin, Deutschland; 8 Department of Rheumatology and Clinical Immunology, Charité Universitätsmedizin Berlin, Berlin, Deutschland; 9 Department of Gastroenterology, Hepatology, Infectious Diseases and Rheumatology, Ernst von Bergman Hospital Potsdam, Potsdam, Deutschland; 10 HMU Health and Medical University Potsdam, Potsdam, Deutschland; 11 Klinik für Rheumatologie, Universitätsklinikum Düsseldorf, Düsseldorf, Deutschland

**Keywords:** Arbeitszufriedenheit, Chancengleichheit, Fachkräftemangel, Mitarbeiterbindung, Arbeitsmodelle, Job satisfaction, Equal opportunities, Staff shortage, Workforce retention, Working models

## Abstract

**Hintergrund:**

Der demografische Wandel und der zunehmende Fachkräftemangel im Gesundheitswesen stellen das deutsche Gesundheitssystem vor große Herausforderungen. Die Rheumatologie ist davon aufgrund der steigenden Patientennachfrage und der sich wandelnden gesellschaftlichen Erwartungen hinsichtlich Work-Life-Balance, Familienvereinbarkeit und Gendergerechtigkeit besonders betroffen. Infolgedessen gewinnen flexible Arbeitszeitmodelle an Bedeutung.

**Ziel:**

Ziel war die Erhebung bestehender und gewünschter Arbeitszeitmodelle sowie deren Umsetzbarkeit und Hürden aus Sicht rheumatologisch tätiger Mitarbeitender und Führungskräfte.

**Material und Methoden:**

Die Kommission Chancengleichheit der DGRh entwickelte zwei Online-Fragebögen für Mitarbeitende und Leitende, die die aktuelle Beschäftigungssituation, bestehende flexible Arbeitszeitmodelle sowie Präferenzen und Machbarkeit verschiedener Arbeitszeitmodelle abfragten. Die Umfrage wurde zwischen September 2024 und März 2025 über den Verteiler der DGRh, des Verbands Rheumatologischer Akutkliniken (VRA) sowie über persönliche Kontakte per Mail verbreitet und anschließend deskriptiv ausgewertet.

**Ergebnisse:**

Insgesamt nahmen 151 Personen teil (111 Mitarbeitende, 40 Leitende). Teilzeitarbeit, insbesondere ambulant, war verbreitet und wurde häufig gewünscht. Die 4‑Tage-Woche und Homeoffice wurden als attraktiv bewertet, aber als organisatorisch herausfordernd eingeschätzt. 24 % der Mitarbeitenden wechselten bereits wegen fehlender Flexibilität den Arbeitsplatz, 30 % der Leitenden verloren dadurch Mitarbeitende. Die Mehrheit priorisiert Flexibilität bei gleicher Arbeitszeit über Arbeitszeitverkürzung bei geringerer Flexibilität.

**Diskussion:**

Flexible Arbeitszeitmodelle sind gewünscht und oft umsetzbar, erfordern jedoch individuelle Lösungen und strukturelle Anpassungen. Sie sind entscheidend für Zufriedenheit und Bindung von Fachkräften und sollten strategisch gefördert werden, um die rheumatologische Versorgung langfristig zu sichern.

## Abstract (en)

**Background:**

Demographic change and a growing shortage of healthcare professionals pose significant challenges to the German healthcare system. Rheumatology is particularly affected due to increasing patient demand and evolving societal expectations regarding work–life balance, family compatibility, and gender equity. As a result, flexible working time models are gaining importance.

**Objective:**

This study aimed to assess existing and desired working time models in rheumatology, their feasibility, and perceived barriers from the perspectives of both employees and leadership.

**Methods:**

The Commission of Equal Opportunities of the German Society for Rheumatology (DGRh) developed two online questionnaires targeting rheumatology healthcare professionals in employee and leadership roles asking about their current employment situation, existing flexible working time models, and preferences and feasibility of various working time models. The survey was distributed between September 2024 and March 2025 via the mailing lists of the DGRh and the Association of Rheumatological Acute Clinics (VRA), and via personal contacts by email, and subsequently evaluated descriptively.

**Results:**

A total of 151 individuals participated (111 employees, 40 leaders). Part-time work, especially in outpatient care, was common and frequently desired. The 4‑day workweek and hybrid models (e.g., home office) were considered attractive but posed organizational challenges. Of the employees, 24% reported having changed jobs due to a lack of flexible options; 30% of leaders had lost staff for the same reason. Most respondents prioritized flexibility over reduced working hours.

**Conclusion:**

Flexible working time models are generally desired and often feasible in rheumatology but require tailored implementation and structural support. They are essential for improving job satisfaction and staff retention and should be strategically promoted to ensure sustainable rheumatologic care.

## Hintergrund

Der demografische Wandel und der zunehmende Fachkräftemangel stellen das deutsche Gesundheitswesen vor wachsende Herausforderungen. Besonders betroffen ist die Rheumatologie, ein Fachgebiet mit hoher Spezialisierung und zugleich steigender Patientennachfrage infolge der Zunahme chronischer entzündlich-rheumatischer Erkrankungen [1, 2]. Gleichzeitig gewinnen gesellschaftliche Themen wie die Vereinbarkeit von Familie und Beruf, veränderte Erwartungen an die *Work-Life-Balance *sowie der *Gender Pay Gap* [3] zunehmend an Bedeutung für die arbeitende Bevölkerung. Dies beeinflusst auch die Arbeitssituation für Ärzt:innen in der Rheumatologie, die sich in zunehmender Teilzeittätigkeit und einem häufigeren Angestelltenverhältnis in Versorgungszentren und Praxen widerspiegelt [1]. Nicht nur, da die Rheumatologie sowohl international als auch in Deutschland zunehmend weiblicher wird [1, 4], sondern auch im Kontext der Erwartungen der sog. Millennials [5], gewinnen zukunftsfähige Arbeitsbedingungen für rheumatologisch tätige Ärzt:innen verstärkt an Bedeutung. Insbesondere müssen diese Bedingungen die Vereinbarkeit von Familie und Beruf unter Berücksichtigung von Mutterschutz, Elternzeit sowie Care-Arbeit für alle Geschlechter stärker in den Fokus rücken. Vor diesem Hintergrund rücken flexible Arbeitszeitmodelle zunehmend in den Mittelpunkt – sowohl als Mittel zur Erhöhung der Attraktivität rheumatologischer Tätigkeiten als auch zur Sicherstellung einer kontinuierlichen und qualitativ hochwertigen Versorgung der Patient:innen.

Während in einigen Fachbereichen der Medizin und in der Pflege [6–9] bereits Erfahrungen und Präferenzen hinsichtlich flexibler Arbeitszeiten untersucht wurden, besteht eine Forschungslücke in Bezug auf die Ausgestaltung flexibler Arbeitszeitmodelle in der Rheumatologie. In einer bundesweiten Umfrage der Medizinischen Hochschule Hannover gaben die in der Rheumatologie tätigen Ärzt:innen ein starkes Missverhältnis zwischen tatsächlicher und gewünschter Arbeitszeit an und stuften ihre Arbeitsbelastung mehrheitlich als hoch oder sehr hoch ein [10]. Eine weitere Umfrage unter Weiterbildungsassistent:innen in der Rheumatologie in Sachsen ergab, dass 48 % der Befragten in Teilzeit als Rheumatolog:in arbeiten möchten. Auch hier wurde die Vereinbarkeit von Familie und Beruf als sehr relevant für das künftige Berufsleben angegeben [11]. Flexibilität und Freiwilligkeit haben sich in der Pflege als entscheidende Faktoren für die Steigerung der Arbeitszufriedenheit herausgestellt [9]. In einer Umfrage der Deutschen Gesellschaft für Rheumatologie und klinischen Immunologie e. V. (DGRh) gaben Assistenzärzt:innen in der rheumatologischen Weiterbildung der Vereinbarkeit von Familie/Privatleben und der beruflichen Tätigkeit die höchste Priorität [12]. Die flexible Gestaltung von Arbeitszeiten, Homeoffice, weniger Überstunden und eine bessere Planung von Arbeitszeiten wurden als relevante Faktoren zur Verbesserung der Balance zwischen Arbeit und Privatleben angeführt.

Die hier vorgestellte Umfrage wurde durch die Kommission Chancengleichheit der DGRh entwickelt und durchgeführt, um bestehende und gewünschte Arbeitszeitmodelle sowie deren Umsetzbarkeit und mögliche Hürden aus Sicht von Mitarbeitenden und Führungskräften in der rheumatologischen Versorgung in Deutschland zu untersuchen.

## Methoden

Der Fragebogen ist angelehnt an die Befragung der Bundesanstalt für Arbeitsschutz und Arbeitsmedizin [13]. Auf dieser Grundlage entwickelte die Kommission zwei Online-Fragebögen, je einen für in der Rheumatologie tätige Ärzt:innen in Weiterbildung oder Facharzt:innen (FÄ) sowie alle weiteren angestellten Rheumatolog:innen ohne Führungsposition (im Folgenden „Mitarbeitende“) und einen Fragebogen für Rheumatologi:innen in Führungspositionen (Chefärzt:innen, Bereichsleitungen, Sektionsleitungen und Inhaber:innen von Praxen oder medizinischen Versorgungszentren – „Leitende“). Die Entwicklung des Fragebogens erfolgte durch eine Arbeitsgruppe der Kommission mit anschließender Überarbeitung und Konsentierung innerhalb der gesamten Kommission. Beide Fragebögen gliederten sich in einen obligatorischen und einen fakultativen Teil. Der Fragebogen für Mitarbeitende umfasste 46 Fragen (19 obligat, 27 fakultativ) und der für Leitende 42 Fragen (19 obligat, 23 fakultativ).

Abgefragt wurden die aktuelle Beschäftigungssituation, bestehende flexible Arbeitszeitmodelle sowie Präferenzen und Machbarkeit verschiedener Arbeitszeitmodelle.

Folgende Fragen wurden zur Akzeptanz und Umsetzbarkeit von Arbeitszeitmodellen gestellt:

1. Welche Arbeitszeitmodelle gibt es bereits in Ihrer Arbeitsstelle? (Teilzeittätigkeit stationäre Versorgung, Teilzeittätigkeit ambulante Versorgung, Gleitzeit 7–21 Uhr, Homeoffice, Jobsharing stationäre bzw. ambulante Versorgung und 4‑Tage-Woche)
2. Welche dieser Arbeitszeitmodelle halten Sie an Ihrer Arbeitsstelle für umsetzbar? (gut/eher/eher nicht/nicht/kann ich nicht beurteilen)
3. Welche dieser Arbeitszeitmodelle würden Sie sich für Ihre Arbeitsstelle wünschen?
4. Was ist Ihnen bei den Arbeitszeitmodellen wichtig? (Skala von 1–10, 1 = weniger Arbeitszeit bis 10 mehr Flexibilität bei gleicher Arbeitszeit)
5. Worin sehen Sie jeweils limitierende Faktoren für die einzelnen Arbeitszeitmodelle? (Notwendigkeit von Präsenz zur Patientenversorgung, Selbstmanagement, Dienstplanung/Organisation Abteilung, unscharfe Trennung von Arbeitszeit und Freizeit, Sichtbarkeit für Vorgesetzte, Verpassen von Meetings, schlechtere Aufstiegschancen)
6. Hat das vorhandene bzw. fehlende Angebot bestimmter Arbeitszeitmodelle (z. B. Teilzeit, Homeoffice) bei Ihnen schon einmal aktiv zum Stellenwechsel angeregt oder beigetragen bzw. zum Verlust von Mitarbeitenden geführt? (Ja/Nein/Ist mir nicht bekannt)
7. Halten Sie diese flexiblen Arbeitszeitmodelle auch für umsetzbar für eine Führungsposition? (nicht umsetzbar/bis zur oberärztlichen Ebene umsetzbar/bis zur chefärztlichen Ebene umsetzbar)
8. Für Mitarbeitende: Beinhaltet Ihre aktuelle Arbeitszeit aktive Forschungszeit (o. a. Aktivitäten, wie z. B. Berufspolitik, Kommissionsarbeit), d. h. können Sie innerhalb Ihrer vorgesehenen Arbeitszeit forschen? (Ja/Nein/Ist für mich irrelevant) Für Leitende: Sind Sie mit der Durchführung von Forschungsaktivitäten innerhalb der Arbeitszeit einverstanden bzw. ist dies ausdrücklich gewünscht? (Ja, einverstanden/Ja, ausdrücklich gewünscht/Nein, trifft für mich nicht zu)

Der fakultative Part umfasste Abfragen zur persönlichen Situation in Bezug auf die Arbeit. (u. a. Alter, Geschlecht, Bundesland vom Arbeitsort, Dauer der Berufstätigkeit in Jahren, Anzahl Kinder, Auswirkung auf Partnerschaft, Betreuung von Angehörigen). Der vollständige Fragebogen ist online als *Supplementary* verfügbar.

Als Plattform wurde „Question-Pro“ genutzt, welche eine Datenschutz-Grundverordnung(DSGVO)-konforme Datenerhebung ermöglicht. Die Verteilung der Umfrage erfolgte im Zeitraum von September 2024 bis März 2025 über den Newsletter der DGRh (*n* = 1870 ärztliche und nichtärztliche Mitglieder), des Verbands Rheumatologischer Akutkliniken (VRA; *n* = 55 Kliniken) sowie über persönliche Kontakte (ca. *n* = 30). Die genaue Zahl der kontaktierten Ärzt:innen ist nicht festzustellen, da zum einen Personen über verschiedene Kanäle doppelt angeschrieben wurden und der DGRh-Verteiler sowohl ärztliche als auch nichtärztliche Mitglieder umfasst.

Im Anschluss wurden die Ergebnisse mittels deskriptiver statistischer Methoden aufbereitet.

## Ergebnisse

### Beschäftigungsverhältnisse der Befragten

An der Umfrage nahmen insgesamt 151 Personen teil, darunter 111 Mitarbeitende und 40 Leitende. Unter den Mitarbeitenden befanden sich 53 Ärztinnen und Ärzte in Weiterbildung, 23 waren Fachärzt:innen, 6 Funktionsoberärzt:innen, 22 Oberärzt:innen, 5 hatten eine Sektionsleitung inne und 2 waren in leitender Funktion in einer Praxis/MVZ tätig. 55 % waren vollzeitbeschäftigt und 43 % gaben eine Teilzeittätigkeit an. Der überwiegende Teil (99 %) war in einem Angestelltenverhältnis, wobei 54 % ein befristetes Beschäftigungsverhältnis angaben.

Von den Leitenden waren 19 Chefärzt:innen, 11 Sektionsleitende, 8 hatten eine Leitungsposition in einer Praxis oder einem MVZ und 2 waren Oberärzt:innen. Die Teamgröße variierte und betrug bei 43 % über 10, bei 28 % 6–10 und bei 25 % bis zu fünf Mitarbeitende (Tab. 1).

**Tab. 1:** Charakteristika

	Mitarbeitende	Leitende
*Anzahl*	111	40
*In Rheumatologie tätig*	99 % (110/111)	98 % (39/40)
*Position *		
Ärzt:in in Weiterbildung	48 % (53/111)	–
Fachärzt:in	21 % (23/111)	–
Funktionsoberärzt:in	5 % (6/111)	–
Oberärzt:in	20 % (22/111)	5 % (2/40)
Sektionsleitung	5 % (5/111)	28 % (11/40)
Leitung in Praxis/MVZ	2 % (2/111)	20 % (8/40)
Chefärzt:in	–	48 % (19/40)
*Teamgröße *		
*>* *10*	52 % (58/111)	43 % (17/40)
6 bis 10	28 % (31/111)	28 % (11/40)
Bis 5	20 % (22/111)	25 % (10/40)
*Keine Angabe*	0 % (0/111)	5 % (2/40)
*Beschäftigungsverhältnis*		
Vollzeit	55 % (61/111)	65 % (26/40)
Teilzeit bis 50 %	6 % (7/111)	–
Teilzeit 50–75 %	24 % (27/111)	7,5 % (3/40)
Teilzeit > 75 %	12 % (13/111)	12,5 % (5/40)
Trifft für mich nicht zu	1 % (1/111)	15 % (6/40)
Angestellt	99 % (110/111)	70 % (28/40)
Selbstständig	1 % (1/111)	17,5 % (7/40)
Sonstiges	–	12,5 % (5/40)
Befristetes Beschäftigungsverhältnis	54 % (60/111)	–
Optionaler Part*		
*Persönliche Angaben*		
*Alter, MW in Jahren (Anzahl Antworten)*	40 J. (86)	53 J. (33)
20–29 Jahre	8 % (7/86)	–
30–39 Jahre	59 % (51/86)	–
40–49 Jahre	19 % (16/86)	33 % (13/39)
50–59 Jahre	5 % (4/86)	26 % (10/39)
60+	9 % (8/86)	26 % (10/39)
*Berufstätig in Jahren, MW*	12 J. (84)	27 J. (33)
*Weiblich (%)*	70 % (60/86)	30 % (10/33)
*Bundesland*	27 % NRW (23/86) 15 % BW (13/86) 14 % Bayern (12/86) 13 % Berlin (11/86) 8 % Hamburg (7/86) 7 % Hessen (6/86)	18 % Bayern (6/33) 15 % NRW (5/33) übrige < 5
*Arbeitsort ländlich*	9 % (8/85)	27 % (9/33)
*Partnerschaft*	89 % (73/82)	90 % (28/31)
*Kinder*	59 % (48/81)	90 % (26/29)
*Versorgung/Betreuung oder Pflege von Angehörigen*	24 % (19/80)	–

*Da fakultativ, variiert die Anzahl der Beantwortungen

### Bestehende Arbeitszeitmodelle und Wunschmodelle

**Abb. 1:**
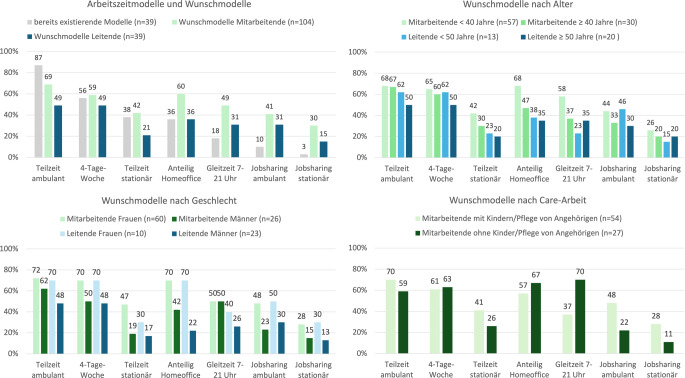
Existierende und gewünschte Arbeitszeitmodelle von Mitarbeitenden und Leitenden, nach Alter, Geschlecht und Care-Arbeit (Kinder und/oder Betreuung bzw. Pflege von Angehörigen)

An den Arbeitsstellen der Leitenden gab es am häufigsten Teilzeittätigkeiten im ambulanten Bereich (87 %), gefolgt von der 4‑Tage-Woche (56 %), Teilzeit im stationären Bereich (38 %) und einem anteiligen Homeoffice (36 %), selten waren Gleitzeit (18 %) und Jobsharing (10 % ambulant und 3 % stationär; Abb. 1).

Die am häufigsten genannten Wunschmodelle waren Teilzeittätigkeit in der ambulanten Versorgung (69 % Mitarbeitende vs. 49 % Leitende), die 4‑Tage-Woche (58 % vs. 49 %) und anteiliges Homeoffice (60 % vs. 36 %). Frauen und Männer wünschten sich Teilzeitmodelle, wobei bis auf Gleitzeit alle Modelle von Frauen häufiger als von Männern als Wunschmodelle angegeben wurden. Hervorzuheben ist hier insbesondere das Homeoffice, welches sowohl von weiblichen Leitenden als auch Angestellten häufig angegeben wurde. Mitarbeitende mit Kindern und/oder zu pflegenden Angehörigen wünschten sich häufiger Jobsharing und ambulante Teilzeitarbeit, während Mitarbeitende ohne Care-Arbeit häufiger anteiliges Homeoffice wünschten. Jüngere wünschten sich tendenziell häufiger Teilzeitmodelle als Ältere.

### Umsetzbarkeit

**Abb. 2:**
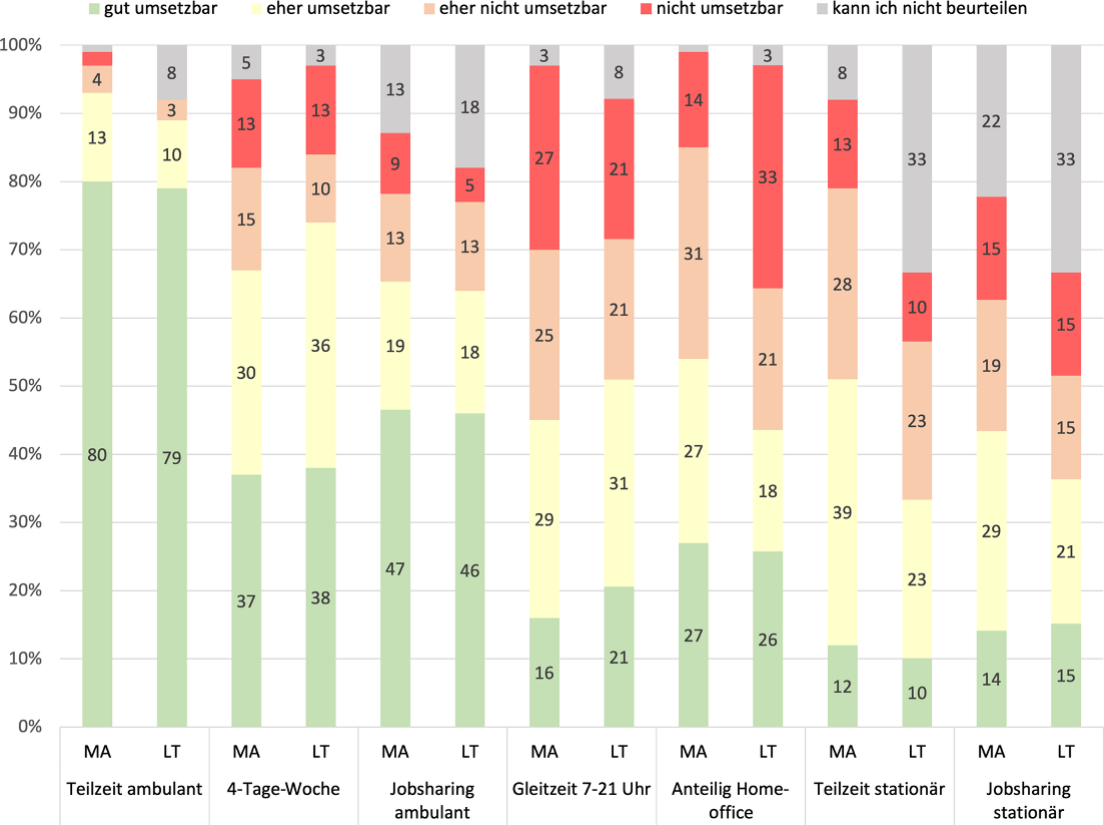
Welche dieser Arbeitszeitmodelle halten Sie an Ihrer Arbeitsstelle für umsetzbar? *MA* Mitarbeitende, *LT* Leitende

Die Umsetzbarkeit von Teilzeittätigkeit in der ambulanten Versorgung wurde von beiden Gruppen überwiegend als gut bewertet (~80 %). Die 4‑Tage-Woche und ambulantes Jobsharing wurden von mehr als der Hälfte beider Gruppen als gut bis eher umsetzbar bewertet. Gleitzeit stuften 52 % der Mitarbeitenden und 42 % der Leitenden als nicht oder eher nicht umsetzbar ein. Beim anteiligen Homeoffice bewerteten 45 % der Mitarbeitenden und 54 % der Leitenden die Umsetzung als schwierig oder nicht möglich. Die Umsetzbarkeit von Teilzeittätigkeit und Jobsharing im stationären Bereich konnten 33 % der Leitenden nicht beurteilen, wurde aber von der Hälfte derjenigen, die es beurteilen konnten, als gut oder eher umsetzbar bewertet. 51 % der Mitarbeitenden schätzten die Teilzeittätigkeit und 43 % das Jobsharing im stationären Bereich für gut bis eher umsetzbar ein (Abb. 2).

### Limitierende Faktoren für die Umsetzbarkeit der Arbeitszeitmodelle

**Abb. 3:**
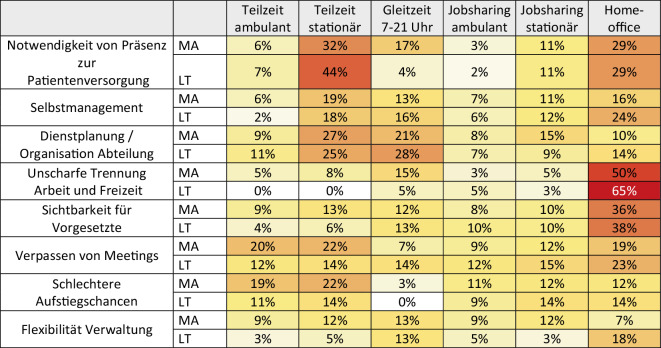
Worin sehen Sie jeweils limitierende Faktoren für die einzelnen Arbeitszeitmodelle? *MA* Mitarbeitende, *LT* Leitende

Als limitierende Faktoren der einzelnen Arbeitszeitmodelle wurden vor allem die notwendige Präsenz bei der Patient:innenversorgung – insbesondere bei Teilzeittätigkeit im stationären Bereich und anteiligem Homeoffice –, organisatorische Herausforderungen bei der Dienstplanung bei stationärer Teilzeit und Gleitzeit sowie die unscharfe Trennung zwischen Arbeitszeit und Freizeit und die reduzierte Sichtbarkeit gegenüber Vorgesetzten im Homeoffice genannt (Abb. 3).

Die Umsetzung von flexiblen Arbeitszeitmodellen bis zur obersten Ebene hielten 49 % der Leitenden für umsetzbar. 24 % der Mitarbeitenden gaben an, dass das fehlende Angebot von flexiblen Arbeitszeitmodellen schon einmal zum Stellenwechsel beigetragen hatte, und 28 % haben darüber nachgedacht, deswegen die Stelle zu wechseln. Bei 10 Leitenden (30 %) hat das fehlende Angebot bestimmter Arbeitszeitmodelle schon einmal zum Verlust von Mitarbeitenden geführt.

### Priorisierung von mehr Flexibilität vs. weniger Arbeitszeit

**Abb. 4:**
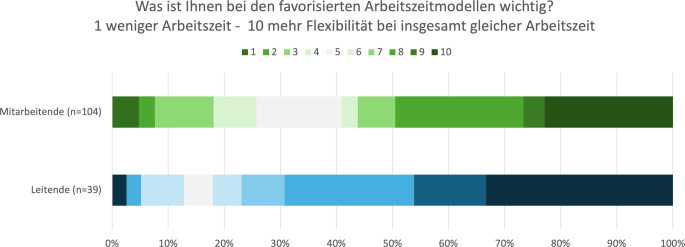
Ergebnisse zur Frage: „Was ist Ihnen bei den Arbeitszeitmodellen wichtig?“ Die Darstellung erfolgt als Heatmap, bei der die Farbsättigung die relative Häufigkeit oder den Anteil der Nennungen widerspiegelt. Ein dunklerer Hintergrund steht für einen höheren Anteil von Befragten, die den jeweiligen Aspekt als wichtig erachten. Helle Flächen weisen entsprechend auf einen geringeren Anteil hin

Insgesamt 82 % der Leitenden und 59 % der Mitarbeitenden priorisierten eine höhere Flexibilität bei insgesamt gleicher Arbeitszeit gegenüber weniger Arbeitszeit (Abb. 4).

### Forschungstätigkeit während der Arbeitszeit

Von den Mitarbeitenden gaben 23 % (20/87) an, dass ihre aktuelle Arbeitszeit auch aktive Forschungstätigkeit beinhaltet, 66 % gaben an, dass ihre Arbeitszeit keine aktive Forschungstätigkeit beinhaltet, und für 11 % war es irrelevant. Von den Leitenden waren 36 % (12/32) mit der Durchführung von Forschungsaktivitäten innerhalb der Arbeitszeit einverstanden, und für 30 % (10/32) war dies ausdrücklich erwünscht.

### Planbarkeit und Mitnahme von Arbeit nach Hause

Die überwiegende Mehrheit der Mitarbeitenden (86 %–73/85) und Leitenden (88 %–28/32) wusste langfristig, wann und wie lange sie arbeiten müssen. Gleichzeitig berichteten 51 % der Mitarbeitenden (42/82), dass sie immer oder oft Arbeit mit nach Hause nehmen, da während der üblichen Arbeitszeit nicht alles zu schaffen sei. 22 % der Leitenden bestätigten, dies bei ihren Mitarbeitenden wahrzunehmen.

### Auswirkung von Arbeitsbelastung/-zeit auf Partnerschaft

Insgesamt 89 % der Mitarbeitenden und 90 % der Leitenden gaben an, in einer Partnerschaft zu leben. Die Partner:innen arbeiteten im Median 41 h/Woche (Mitarbeitende) vs. 31 h/Woche (Leitende). Gemeinsame freie Wochenenden hatten Mitarbeitende und Leitende mehrheitlich 2‑mal im Monat. 23 % der Mitarbeitenden (18/80) und 7 % (2/29) der Leitenden gaben an, dass ihre Arbeitsbelastung und/oder ihre Arbeitszeiten schon einmal wesentlicher Trennungsgrund für eine partnerschaftliche Beziehung war.

## Diskussion

Die Ergebnisse der vorliegenden Erhebung geben einen Einblick in die Beschäftigungsrealität und die Erwartungen an flexible Arbeitszeitmodelle in der Rheumatologie in Deutschland. Die Daten zeigen, dass einige flexible Arbeitszeitmodelle bereits etabliert sind; jedoch besteht bei anderen Modellen – trotz eines klar artikulierten Bedarfs seitens der Mitarbeitenden und Führungskräfte – noch ein erhebliches Potenzial für deren Ausbau. Diese Diskrepanz zwischen Bedarf und tatsächlicher Umsetzung weist auf bestehende strukturelle und organisatorische Herausforderungen hin, die einer breiteren Implementierung entgegenstehen.

Deutlich wird dies am Beispiel der Teilzeittätigkeit in der ambulanten Versorgung, die mit 87 % bereits weit verbreitet ist und gleichzeitig von 69 % der Mitarbeitenden und 49 % der Leitenden weiterhin als wünschenswert benannt wird. Dies spricht für eine hohe Akzeptanz und Relevanz dieses Modells. Auch im stationären Bereich arbeiten bereits 39 % der rheumatologischen Fachärzt:innen in Teilzeit; der Anteil ist seit 2018 um 14 % gestiegen [1]. Demgegenüber stehen Modelle wie Jobsharing oder Gleitzeit, die sowohl in der Umsetzung als auch in der Wahrnehmung ihrer Umsetzbarkeit deutlich zurückfallen. Die 4‑Tage-Woche und anteiliges Homeoffice werden von beiden Gruppen als attraktiv bewertet, sind jedoch bislang nur in etwa der Hälfte der Einrichtungen implementiert. Beim anteiligen Homeoffice mag dies insbesondere damit zusammenhängen, dass es von einem substanziellen Anteil der Antwortenden als nicht umsetzbar bewertet wurde. Die Akzeptanz ist für die 4‑Tage-Woche (4 Arbeitstage bei gleicher oder reduzierter Stundenanzahl und vollem Lohnausgleich) mit 56 % überraschend hoch, was die Frage aufkommen lässt, ob den Teilnehmenden diese Definition der 4‑Tage-Woche bekannt war. Anteiliges Homeoffice wurde bereits von den Weiterbildungsassistent:innen als relevanter Faktor für eine Verbesserung der Vereinbarkeit von Familie und Beruf benannt [12] und wird auch in dieser Umfrage vor allem von Jüngeren und von Frauen als wünschenswert geschätzt, gleichzeitig werden jedoch auch die dabei entstehenden die Einschränkungen für die Patientenversorgung anerkannt. Die Art der Arbeit im anteiligen Homeoffice wurde nicht dezidiert definiert. Hier sind verschiedene Modelle denkbar, wie Arztbriefschreibung und andere administrative Tätigkeiten sowie perspektivisch auch Videosprechstunden.

Das große Interesse an Teilzeitmodellen ist auch in anderen Fachbereichen präsent. In einer aktuellen Umfrage unter Fachärzt:innen für Gynäkologie und Weiterbildungsassistent:innen sprachen sich nur 13 % der Befragten für eine Vollzeitbeschäftigung aus, obwohl 63 % der Weiterbildungsassistent:innen in Vollzeit tätig waren. Deutliche Unterschiede zeigten sich zwischen den Geschlechtern: 40 % der Männer bevorzugten Vollzeitstellen, während nur 11 % der Frauen diese Präferenz teilten [7]. Unsere Umfrage bestätigt, dass sich Frauen und Männer Teilzeitmodelle wünschen, Frauen aber deutlich häufiger als Männer. Studien aus der Allgemeinmedizin und aus der Rheumatologie zeigen, dass Frauen im Durchschnitt weniger Stunden als Männer arbeiten und damit weniger Patient:innen versorgen, allerdings auch mehr Zeit mit ihren Patient:innen während eines Kontakts verbringen und sich mit einer größeren Anzahl verschiedener Probleme bei einem Besuch befassen [4, 14]. Damit trägt die Zunahme der weiblichen Medizin auch zu einem größeren Bedarf an Teilzeitmodellen bei.

Auf der anderen Seite zeigen die Einschätzungen zur Umsetzbarkeit, dass insbesondere Modelle mit höherem organisatorischem Aufwand – wie Gleitzeit oder stationäres Jobsharing – als herausfordernd wahrgenommen werden. Leitende äußerten zudem häufiger Unsicherheit in der Bewertung der Umsetzbarkeit, was auf einen Bedarf an struktureller und rechtlicher Orientierung hinweist. Limitierende Faktoren wie die Notwendigkeit physischer Präsenz, Herausforderungen in der Dienstplanung sowie die Sorge um Sichtbarkeit und Karrierechancen im Homeoffice spiegeln bekannte Spannungsfelder zwischen Flexibilisierung und institutionellen Anforderungen wider.

Besonders relevant ist, dass 24 % der Mitarbeitenden angaben, aufgrund fehlender flexibler Arbeitszeitmodelle bereits den Arbeitsplatz gewechselt zu haben, und 28 % dies zumindest in Erwägung gezogen haben. Auch auf Seiten der Leitenden wurde berichtet, dass das Fehlen entsprechender Angebote bereits zum Verlust von Mitarbeitenden geführt habe (30 %). Dies unterstreicht die strategische Bedeutung flexibler Arbeitszeitgestaltung für die Personalgewinnung und -bindung. Eine Steigerung der Arbeitszufriedenheit ist unabdingbar, um Burnout-fördernde Strukturen zu reduzieren [15] und die Attraktivität unseres Fachbereiches zu erhöhen. In der Umfrage der Medizinischen Hochschule Hannover schätzten 15 % der Rheumatolog:innen ihr aktuelles Risiko, an Stress oder Burnout-Syndromen zu erkranken als sehr hoch und weitere 17 % als hoch ein [10]. Der Verbleib der gut ausgebildeten jungen rheumatologischen Fachärzt:innen ist in Anbetracht der alternden Rheumatologie (von allen berufstätigen Fachärzt:innen für Rheumatologie sind 30 % 60 Jahre und älter [1]) ein entscheidender Faktor für die Sicherung der rheumatologischen Versorgung. Einer Abwanderung in attraktivere Arbeitsbedingungen in anderen Fächern oder auch Branchen (z. B. Industrie) sollte unbedingt entgegengewirkt werden. Wichtig ist dabei, den Wunsch nach Teilzeitmodellen nicht als ursächlich für Ärzt:innenmangel zu betrachten, sondern als Möglichkeit, die Rheumatologie durch das Angebot flexibler Arbeitszeitmodelle attraktiver zu machen [16].

Gerade die Rheumatologie zeichnet sich durch spezifische Rahmenbedingungen aus, die flexible Arbeitszeitmodelle besonders gut ermöglichen könnten. Zum einen ist die Betreuung rheumatischer Erkrankungen häufig durch planbare ambulante Sprechstunden geprägt, die sich gut in flexible Zeitfenster strukturieren lassen. Die Mehrzahl der Patient:innen benötigt regelmäßig wiederkehrende Kontrollen und Therapieanpassungen, wodurch Termine weitgehend im Voraus geplant werden können. Auch ist die Rheumatologie im Vergleich zu anderen Disziplinen weniger von akuten Notfällen betroffen, dies reduziert die Notwendigkeit von Bereitschaftsdiensten und ermöglicht eine gleichmäßigere Arbeitsverteilung. Darüber hinaus ist die interdisziplinäre Zusammenarbeit mit anderen Fachbereichen und therapeutischen Disziplinen oftmals gut koordinierbar, und eine kontinuierliche gemeinsame Präsenz verschiedener Fachrichtungen ist im Vergleich zu chirurgischen Fächern zumeist nicht notwendig. Im Gegensatz dazu steht der Bereich der stationären Versorgung unter der Notwendigkeit einer lückenlosen Betreuung. Eine Abwesenheit bzw. die Möglichkeit, diese Kontinuität zu gewährleisten, kann fehlerhafte Verläufe produzieren, etwa bei Nichteinhaltung oder Gewährleistung entsprechender Fallübergaben oder interdisziplinären Besprechungen bei diesen doch komplexeren Patient:innen verglichen zur Situation der ambulanten Versorgung. Bei der Umsetzung flexibler Arbeitszeitmodelle ist zudem die Akzeptanz von Patient:innen und ambulantem und stationärem Personal zu berücksichtigen und zu evaluieren.

Die Priorisierung von Flexibilität gegenüber einer Reduktion der Gesamtarbeitszeit (82 % der Leitenden, 59 % der Mitarbeitenden) legt nahe, dass es weniger um eine Arbeitszeitverkürzung als vielmehr um eine bessere Anpassung an individuelle Lebensrealitäten geht. Dies eröffnet Handlungsspielräume für Institutionen, die durch gezielte Maßnahmen zur Flexibilisierung – etwa durch Gleitzeitfenster, hybride Arbeitsmodelle oder teamorientierte Dienstplanung – sowohl die Attraktivität des Fachgebiets als auch die Versorgungssicherheit stärken können.

Eine wesentliche Limitation der vorliegenden Studie liegt in der komplexen Rollenverteilung mancher Befragter – insbesondere von Personen in Sektionsleitungspositionen. Da diese Personen sowohl als Arbeitnehmer:innen als auch als Arbeitgeber:innen fungieren, besteht die Möglichkeit, dass sie Fragen aus beiden Perspektiven beantworteten. Dies kann eine Trennschärfe der Daten beeinträchtigen und zu Überschneidungen oder Widersprüchen in den Antworten führen. Infolgedessen könnte die Differenzierung zwischen den verschiedenen Rollen und deren Einfluss auf die Einstellungen zu flexiblen Arbeitszeitmodellen nicht klar herausgearbeitet werden. Ein weiteres methodisches Problem ist die oben bereits genannte fehlende Angabe einer Definition der Arbeitszeitmodelle in unserer Umfrage, weshalb nicht klar ist, ob alle Teilnehmenden z. B. das Konzept der 4‑Tage-Woche im Fragebogen einheitlich und in der intendierten Weise verstanden haben. Die fehlende Standardisierung oder genauere Erläuterung dieses Arbeitszeitmodells im Fragebogen könnte zu unterschiedlichen Interpretationen geführt haben, wobei wir davon ausgehen, dass den Teilnehmenden alle anderen Arbeitszeitmodelle bekannt sein sollten. Dennoch sollten diese Faktoren bei der Interpretation der Ergebnisse berücksichtigt werden. Die Verbreitung der Umfrage über einen gemischten ärztlichen und nichtärztlichen Verteiler und persönliche Kontakte führt dazu, dass keine eindeutige Aussage zur Antwortfrequenz getätigt werden kann.

Die finanziellen Konsequenzen flexibler Arbeitszeitmodelle wurden explizit nicht in diese Umfrage aufgenommen. Es sollte den Teilnehmenden bewusst sein, dass eine Arbeitszeitreduktion mit Gehaltseinbußen einhergeht (Ausnahme 4‑Tage-Woche nach der landläufigen Definition, s. oben). Für Kliniken und Praxen sollten keine relevanten finanziellen Konsequenzen entstehen, da durch Arbeitszeitreduktionen Arbeitsstellen frei werden, die entsprechend besetzt werden können. Die Umsetzung flexibler Arbeitszeitmodelle sollte nicht dazu führen, dass weniger Patient:innen versorgt werden.

## Fazit für die Praxis

Flexible Arbeitszeitmodelle sind grundsätzlich erwünscht und meist umsetzbar, stellen aber organisatorische Herausforderungen dar. Eine gezielte Implementierung trägt maßgeblich zur Zufriedenheit und Bindung der Mitarbeitenden bei und sollte daher (wenn möglich) umgesetzt werden. Ein übergeordnetes Arbeitszeitmodell für alle erscheint hierbei nicht sinnvoll, vielmehr sind individuelle Modelle, die auf die jeweiligen Bedürfnisse der Beteiligten und auf die Voraussetzungen der jeweiligen Einrichtung abgestimmt sind, anzustreben. Dies gilt sowohl für ambulante als auch für stationäre Konzepte, wobei Letzteres aufgrund der Sicherstellung der Patientenversorgung über den gesamten Tag eine besondere Herausforderung darstellt. In einem nächsten Schritt möchten wir die Patient:innen befragen, wie gut sie mit den Konsequenzen von Teilzeitmodellen zurechtkommen und welche Wünsche (z. B. kontinuierliche Betreuung durch die gleiche Ärzt:in, flexiblere Sprechstundenzeiten) hierbei in Zukunft zu berücksichtigen sind. Wichtig ist, dass die Qualität der Patientenversorgung durch die Umsetzung flexibler Arbeitszeitmodelle nicht etwa leidet, sondern im Gegenteil durch eine höhere Zufriedenheit der Behandelnden das Potenzial hat, sich noch weiter zu verbessern.

## Data Availability

Die Daten werden auf begründeten Antrag zur Verfügung gestellt.
